# Daily Physical Activity and Screen Time, but Not Other Sedentary Activities, Are Associated with Measures of Obesity during Childhood

**DOI:** 10.3390/ijerph120100146

**Published:** 2014-12-23

**Authors:** Shoo Thien Lee, Jyh Eiin Wong, Safii Nik Shanita, Mohd Noor Ismail, Paul Deurenberg, Bee Koon Poh

**Affiliations:** 1 School of Healthcare Sciences, Faculty of Health Sciences, Universiti Kebangsaan Malaysia, 50300 Kuala Lumpur, Malaysia; E-Mails: shoothien@hotmail.com (S.T.L.); wjeiin@ukm.edu.my (J.E.W.); nikshanita@ukm.edu.my (S.N.S.); 2 School of Hospitality, Tourism and Culinary Arts, Taylor’s University, Subang Jaya 47500, Malaysia; E-Mail: ismailnoor49@gmail.com; 3 Nutrition Consultant, Telaga Harbour Park, Lot 1, 07000 Langkawi, Malaysia; E-Mail: paul.deurenberg@gmail.com; 4 National Coordinator (Malaysia), the SEANUTS Study Group, 50300 Kuala Lumpur, Malaysia

**Keywords:** anthropometric indicators, body fat, body mass index, physical activity, screen time, sedentary behaviour, waist circumference

## Abstract

Childhood obesity is related to low physical activity level and a sedentary lifestyle. The aim of this study was to assess the physical activity level and sedentary behaviour of Malaysian children aged 7 to 12 years and to examine their association with body mass index (BMI), BMI-for-age Z-score (BAZ), body fatness (%BF) and waist circumference (WC). A total of 1736 children, representing all ethnic groups were recruited from six regions of Malaysia. Anthropometric measurements included body weight, height and waist circumference. Body fat percentage (%BF) was assessed using bioelectrical impedance. Physical activity was assessed by a physical activity questionnaire (PAQ) in all children and by pedometers in a subsample (*n* = 514). PAQ score and pedometer step counts were negatively associated with BMI, BAZ, %BF and WC after adjusting for covariates. Screen time was positively associated with BAZ and WC. However, other sedentary activities were not significantly related with any anthropometric indicators. Strategies to promote active living among children in Malaysia should focus not only on increasing physical activity but also emphasise reduction in sedentary behaviours.

## 1. Introduction

The high prevalence of childhood obesity is a health problem worldwide, especially in the developed countries; while in developing countries, it is an emerging problem where the rates of overweight and obesity are catching up with the trends seen in developed nations [1]. In Malaysia, the prevalence of overweight and obesity among children aged 6 to 12 years old increased from 20.7% in year 2002 to 26.4% in year 2008 [2]. Another study conducted in years 2010–2011 in six regions of Malaysia reported 14.4% overweight and 20.1% obesity among urban children aged 7 to 12 years old [3].

Childhood overweight and obesity results in immediate and long-term risks to the health of children [4,5]. According to Wee *et al.* [6], Malaysian children aged 9 to 12 years who were overweight or obese had 16.3 times higher risk of developing metabolic syndrome compared to normal weight children. Overweight and obese children are also at higher risk of developing into obese adults with all its associated non-communicable diseases [7].

Systematic reviews report that overweight and obesity in children are related to lower physical activity level and higher sedentary behaviour [8,9,10]. The average time Malaysian children aged between 9 and 10 years spent in moderate and vigorous physical activity was reportedly between 442 and 490 min per week [11], which approximates the recommended 60 min of moderate intensity physical activity daily [12]. Sedentary behaviour is defined as any activity with energy expenditure of 1.0–1.5 metabolic equivalent units (METs) [13]. A study conducted in a district in Selangor, Malaysia reported that more than half of adolescents aged 13 to 15 years had reported predominantly sedentary lifestyles [14], however little is known about the sedentary behaviour of Malaysian children.

There is a dearth of information on physical activity level, sedentary behaviour as well as achievements of physical activity and screen time recommendations among primary school children in Malaysia. Because physical activity and sedentary behaviour may co-exist, there is a need to understand how each of these components is related to different measures of obesity, independently of socio-demographic and energy intake covariates. Such findings are paramount in providing the basis of evidence for developing intervention strategies to address the escalating problem of childhood obesity in Malaysia. The relationship between physical activity and sedentary behaviour with obesity appears to be a knowledge gap in tackling the dramatic rise of childhood obesity in Malaysia. Hence, this study aimed to investigate the associations between physical activity and sedentary behaviour of children with BMI, BMI-for-age Z-score (BAZ), body fat percentage (%BF), and WC using a nationally representative sample.

## 2. Methodology

### 2.1. Study Design

This cross-sectional study was part of the South East Asian Nutrition Survey (SEANUTS), a multicentre study that was simultaneously conducted among 16,744 children aged 0.5 to 12 years in four countries, namely Indonesia, Malaysia, Thailand and Vietnam [15]. This project was registered in the Netherlands Trial Registry as NTR2462.

In Malaysia, children aged 7 to 12 years were recruited from primary schools from six regions across the country using stratified random sampling [3]. The study was conducted according to the Guidelines of the Declaration of Helsinki and the study protocol was approved by the Medical Research Ethics Committee of Universiti Kebangsaan Malaysia (Project code: NN-072-2009). Written informed consent from the parents and verbal assent from the children was obtained before measurements.

Subjects comprised boys and girls, from both urban and rural areas, and from the main ethnic groups in Malaysia, namely Malays, Chinese, Indians as well as Sabah and Sarawak *bumiputra*. Out of the 3542 subjects participating in SEANUTS Malaysia, 1971 subjects were aged 7 to 12 years old. Children who reported sedentary activity time of more than 12 h per day were excluded from analysis, after taking into account the time spent in school and for sleeping (*n* = 235). In this paper, a total of 1736 children (823 boys; 913 girls), which encompasses a subsample of 514 children (216 boys; 298 girls) with pedometer data, were included in the analysis.

### 2.2. Physical Activity and Sedentary Behaviour

Physical activity was measured with a subjective method employing the Physical Activity Questionnaire for Children (PAQ-C) adapted from Crocker *et al.* [16]. The questionnaire is a validated Malay version of the PAQ-C with good internal consistency and acceptable validity [17]. Children were required to complete 10 questions related to their daily physical activity over the previous seven days. The first item included the type and frequency of sports during the past seven days. The second to eighth items assessed activity during physical education (PE) classes, recess time, lunch time, after school, evenings, weekend and leisure time. A Likert scale of one to five points was used for these items, ranging from low (score = 1) to high (score = 5). Item 9 was the frequency of engaging in physical activity in last week and item 10 required the children to report unusual activities in the previous week which had not been recorded in items 1 to 9. The PAQ score is the average score of these 10 questions.

A random sub-sample of the children was also objectively measured using a pedometer (Digi-walker CW-700; Yamax) for seven consecutive days. Children were instructed to wear the pedometer at all times except during sleep, shower/bathe and swimming. A weighed average daily step count was calculated from both weekdays and weekend days. The pedometer step counts were considered valid if two criteria were fulfilled: (1) the weighted average step counts had to be more than 1000 steps per day [18]; and (2) the period that the pedometer was worn had to be at least 10 h per day [19]. A minimum number of valid days of at least 3 weekdays and 1 weekend day was used for the calculation of average pedometer step counts [19]. A cut-off value of 13,000 steps per day for boys and 11,000 steps per day for girls was used as recommended pedometer step counts [20].

Sedentary behaviour of the children was assessed with a questionnaire adapted from the Child and Adolescent Physical Activity and Nutrition Survey (CAPANS) [21]. Children were required to complete the questions related to their daily sedentary activities over the past seven days. The type of sedentary activities included watching television, playing video games, surfing internet/using computer, doing homework/revisions, attending extra classes (not within regular school hours), reading, and sitting while playing. The weighted average time spent watching television, playing video games and using computer were calculated from that reported for weekdays and weekend days. The sum of these weighted average variables forms the “screen time” variable in this study. “Non-screen time” included other sedentary activities besides screen time activities. The recommended screen time level of not more than two hours per day from the American Academy of Pediatrics [22] was used in this study.

### 2.3. Anthropometric Measurements

Height was measured with a wall-mounted stadiometer (SECA model 213, Hamburg, Germany) to the nearest 0.1 cm. Weight was measured to the nearest 0.1 kg with a digital scale (SECA model 803,). Body mass index (BMI, kg/m^2^) was calculated by dividing weight (kg) by the square of height (m). BMI-for age Z-score (BAZ) was calculated with the WHO AnthroPlus software [23]. Children were classified as thin, normal weight, overweight or obese with the cut off of ≤−2SD, >−2SD to <1SD, ≥1SD to <2SD, and ≥2SD, respectively based on the WHO Growth Reference [24].

Waist circumference (WC) was measured at the midpoint between the lowest rib and iliac crest using a Lufkin tape (Model W606PM; Cromwell, UK). Following a standardised protocol by World Health Organization [25], WC was measured to the nearest 0.1 cm. Body fat percentage (%BF) was measured by bioelectrical impedance (Bodystat model 1500 MDD, Isle of Man, UK) using the incorporated formula in the instrument. Subjects were required to fast for at least 8 h and all objects that may interfere with readings were removed before measurements were taken while lying supine for 5 to 10 min.

### 2.4. Confounders

Socio-demographic variables, including age, sex, ethnicity, household income and parental education level were obtained through a parent-administered questionnaire. Energy intake of children was obtained from a validated food frequency questionnaire (FFQ), which comprised 13 food groups and 94 food items [3].

### 2.5. Data Analysis

Complex sample analyses was conducted using SPSS version 18.0 (IBM Corporation, Chicago, IL, USA), using weight factors based on the population census 2010 [26]. The weight factor in the analysis with pedometer counts was adapted to the smaller sample size. Descriptive analysis was used to analyse the children’s characteristics, nutritional status, physical activity score, daily step counts, time spent on sedentary activities and screen time. Differences between the sexes and areas of residence in these parameters were analysed using *t*-test. Logistic regression was used to analyse differences in the percentage of children not meeting recommended pedometer step counts and existing screen time recommendations among sex, area of residence and BMI groups. Generalised Linear Models were applied to determine BMI groups, %BF and WC by daily physical activity and sedentary behaviour, after correcting for age, sex, area of residence, energy intake, ethnicity, household income, and parental education level. Values are expressed as mean ± SE, unless otherwise stated. The significance level was set at *p* < 0.05.

## 3. Results

A total of 1736 subjects, with a subsample of 514 assessed with pedometer, participated in the study. Two different weight factors were used in order to ensure that the results are representative of the estimated population of 2.96 million Malaysian children aged 7 to 12 years old. Descriptive statistics by socio-demographic characteristics and energy intake of children are reported in Table 1. The majority of the parents completed secondary education.

**Table 1:** Socio-demographic characteristics and energy intake of children.

Variables	Total		
	*n* (%)	Mean	SE
Age (years)		10.0	0.1
Energy intake (kcal)		1970	14
Household income (Ringgit Malaysia)		3284	104
**Sex**			
Boys	823 (47.4)		
Girls	913 (52.6)		
**Area of residence**			
Urban	1035 (59.6)		
Rural	701 (40.4)		
**Ethnicity**			
Malay	976 (56.2)		
Chinese	368 (21.2)		
Indian	127 (7.3)		
Others	265 (15.3)		
**Maternal education**			
Non-schooling	21 (1.2)		
Primary	116 (6.7)		
Secondary	1135 (65.4)		
Tertiary	464 (26.7)		
**Paternal education**			
Non-schooling	21 (1.2)		
Primary	126 (7.3)		
Secondary	1100 (63.4)		
Tertiary	489 (28.2)		

Table 2 shows the nutritional status of children. The BMI, BAZ, %BF and WC of urban children were significantly higher than that of rural children. Boys had significantly higher WC than girls in both urban and rural areas. In contrast, %BF of girls, especially in urban areas, was significantly higher than that of boys. Boys had significantly higher BAZ than girls in rural area.

**Table 2:** Nutritional status of children by sex and area of residence.

	Boys		Girls		Total	
	Mean	SE	Mean	SE	Mean	SE
**Urban**						
Sample size	490		545		1035	
Height (cm)	134.8 ^††^	0.4	136.2	0.3	135.7 ^¥¥^	0.3
Weight (kg)	34.2	0.6	34.5	0.6	34.5 ^¥¥^	0.4
BMI (kg/m^2^)	18.3	0.2	18.1	0.2	18.2 ^¥^	0.2
BAZ	0.39	0.10	0.19	0.09	0.29 ^¥^	0.07
BF (%)	24.8 ^†††^	0.6	28.0	0.5	26.4 ^¥^	0.4
WC (cm)	62.7 ^††^	0.7	60.3	0.6	61.6 ^¥¥^	0.5
**Rural**						
Sample size	333		368		701	
Height (cm)	135.5	0.6	134.3	0.6	134.2	0.4
Weight (kg)	33.7	0.8	31.9	0.9	32.3	0.6
BMI (kg/m^2^)	18.0	0.3	17.2	0.3	17.5	0.2
BAZ	0.24 ^†^	0.12	−0.21	0.13	0.01	0.09
BF (%)	23.9 ^†^	0.8	25.9	0.6	25.0	0.5
WC (cm)	61.6 ^††^	0.9	57.9	0.7	59.4	0.6
**All**						
Sample size	823		913		1736	
Height (cm)	134.5 *	0.3	135.4	0.3	134.9	0.2
Weight (kg)	33.5	0.5	33.3	0.5	33.4	0.4
BMI (kg/m^2^)	18.0	0.2	17.7	0.2	17.9	0.1
BAZ	0.28 *	0.08	0.03	0.08	0.15	0.05
BF (%)	24.2 ***	0.5	27.2	0.4	25.7	0.3
WC (cm)	61.8 **	0.6	59.2	0.5	60.5	0.4

BMI: Body Mass Index; BAZ: Body Mass Index-for age Z-score; BF: Body fat; WC: Waist circumference; Mean values were significantly different between the sexes, after adjusting for age and area of residence at * *p* < 0.05, ** *p* < 0.01, *** *p* < 0.001; Mean values were significantly different between area of residence, after adjusting for age and sex at ^¥^ *p* < 0.05, ^¥¥^ *p* < 0.01; Mean values were significantly different between the sexes within area of residence, after adjusting for age at ^†^ *p* < 0.05; ^††^ *p* < 0.01; ^†††^ p < 0.001.

Table 3 shows that the average physical activity score and daily step counts of the children were 2.52 ± 0.02 and 9023 ± 177 steps, respectively. The children spent on average 6.7 ± 0.1 h on sedentary activities daily, of which 3.1 ± 0.1 h was spent on screen time. Boys were more physically active and had higher screen time than girls regardless of the area of residence (*p* < 0.05). The time spent on non-screen sedentary activities, such as revision, attending extra classes, reading, and sitting while playing, were higher in girls as compared to boys in rural area. As shown in Table 3, overweight and obese children are significantly less active than their counterparts, as evidenced in their lower physical activity score and pedometer step counts. However, there are no differences in screen time, non-screen time and total sedentary activities between the two BMI groups.

**Table 3:** Physical activity and time spent on sedentary activities of children according to sex, residential area and BMI status.

	PAQ Score		Pedometer Step Counts (Steps)		Screen Time (Hours)		Non-Screen Sedentary Activities (Hours)		Total Sedentary Activities (Hours)	
	Mean	SE	Mean	SE	Mean	SE	Mean	SE	Mean	SE
Boys	2.62 ***	0.03	9708 ***	284	3.3 ***	0.1	3.5 *	0.1	6.8	0.1
Girls	2.41	0.03	8339	254	2.8	0.1	3.8	0.1	6.6	0.1
Urban children	2.49	0.02	8672 ^¥^	247	3.1	0.1	3.7	0.1	6.7	0.1
Boys	2.61 ^††^	0.03	9370 ^††^	376	3.3 ^†^	0.1	3.6	0.1	6.9	0.1
Girls	2.39	0.03	7966	323	2.9	0.1	3.8	0.1	6.6	0.1
Rural children	2.54	0.03	9375	251	3.1	0.1	3.6	0.1	6.7	0.1
Boys	2.63 ^††^	0.04	10017 ^††^	403	3.4 ^†^	0.1	3.4 ^†^	0.1	6.7	0.2
Girls	2.42	0.05	8786	250	2.8	0.1	3.8	0.1	6.7	0.2
Thinness/normal weight children	2.54 ^γ^	0.02	9326 ^γγγ^	205	3.0	0.1	3.6	0.1	6.6	0.1
Boys	2.64 ^###^	0.03	10097 ^###^	347	3.2 ^##^	0.1	3.5	0.1	6.7	0.1
Girls	2.44	0.03	8487	233	2.8	0.1	3.7	0.1	6.5	0.1
Overweight/ obese children	2.46	0.03	8271	365	3.2	0.1	3.7	0.1	6.9	0.2
Boys	2.59 ^###^	0.04	8492	423	3.5 ^##^	0.2	3.5	0.2	7.0	0.2
Girls	2.33	0.04	8161	526	2.9	0.2	3.9	0.2	6.8	0.2
Total	2.52	0.02	9023	177	3.1	0.1	3.6	0.1	6.7	0.1

Mean values were significantly different between the sexes, after adjusting for age and area of residence at * *p* < 0.05; *** *p* < 0.001; Mean values were significantly different between areas of residence, after adjusting for age and sex at ^¥^ *p* < 0.05; Mean values were significantly different between the sexes within area of residence, after adjusting for age at ^†^ *p* < 0.05; ^††^ *p* < 0.01; Mean values were significantly different between BMI groups, after adjusting for age, sex and area of residence at ^γ^ *p* < 0.05; ^γγγ^ *p* < 0.001; Mean values were significantly different between the sexes within BMI groups, after adjusting for age and area of residence at ^##^ *p* < 0.01; ^###^ *p* < 0.001.

The majority of the children (84.8%) did not meet the recommendation for daily pedometer step counts of 13,000 steps for boys and 11,000 steps for girls (Table 4). A high percentage of overweight and obese children compared to normal weight and thin children did not achieve the recommended daily step counts. Overall, 68.4% of the children exceeded the recommendations of maximally two hours of screen time per day. More girls than boys, and more children from rural than urban areas, achieved screen time recommendations (*p* < 0.05). Boys had longer screen time, but they also had higher PAQ score and pedometer step counts.

**Table 4:** Percentage of children not meeting pedometer steps counts recommendation and those exceeding screen time recommendation according to sex, residential area and BMI status.

	Not Meeting Recommended Pedometer Step Counts		Screen Time More Than 2 h	
	*n*	%	*n*	%
Boys	184	85.1	608	73.9 ***
Girls	252	84.4	575	63.0
Urban children	243	85.8	707	68.3
Boys	94	86.5	358	73.1 ^††^
Girls	148	85.1	346	63.5
Rural children	187	80.8	484	69.1
Boys	86	80.4	256	77.0 ^††^
Girls	101	81.3	224	61.0
Thinness/normal weight children	301	82.4	806	66.8
Boys	124	81.3	386	71.1 ^#^
Girls	178	83.7	416	62.7
Overweight/obese children	134	90.0	382	72.0
Boys	61	95.4	222	79.2 ^##^
Girls	73	85.9	159	63.5
Tota	436	84.8	1187	68.4

Mean values were significantly different between the sexes based on complex sampling logistic regression, after adjusting for age and area of residence at *** *p* < 0.001; Mean values were significantly different between the sexes within area of residence based on complex sampling logistic regression, after adjusting for age and sex at ^††^ *p* < 0.01; Mean values were significant different between the sexes within BMI group based on complex sampling logistic regression, after adjusting for age and area of residence at ^#^ *p* < 0.05; ^##^ *p* < 0.01.

The regression model in Table 5 shows that both subjectively and objectively measured physical activity of urban children (PAQ-score and pedometer step counts) are negatively associated to BMI, BAZ, %BF and WC after adjusting for age, sex, area of residence, ethnicity, energy intake, household income and parental education level. Every one unit increment in PAQ-score results in 0.8 kg/m^2^ lower BMI, while every 1000 steps/day increment results in 0.3 kg/m^2^ lower BMI. Similarly, one unit higher in PAQ-score results in 2.4% lower body fat and 2.3 cm lower WC. Screen time was positively associated with BAZ and WC. An hour longer of screen time results in 0.06 point increment in BAZ and 0.4 cm higher WC. Non-screen sedentary activities were not associated with BMI, BAZ, %BF, and WC.

**Table 5:** Regression models of nutritional status of children based on daily physical activity and sedentary activities.

	PAQ Score		Pedometer Step Counts (Steps)		Screen Time (Hours)		Non-Screen Time Sedentary Activities (Hours)		Total Sedentary Activities (Hours)	
	β	SE	β	SE	β	SE	β	SE	β	SE
Urban										
BMI (kg/m^2^)	−0.82 **	0.26	−0.33 ***	0.09	0.18	0.10	0.02	0.09	0.10	0.07
BAZ	−0.28 **	0.10	−0.09 **	0.03	0.06	0.04	0.01	0.03	0.03	0.03
BF (%)	−2.36 ***	0.55	−0.59 **	0.19	0.36	0.20	−0.14	0.19	0.10	0.15
WC (cm)	−2.33 ***	0.67	−0.86 ***	0.24	0.46	0.26	−0.09	0.22	0.18	0.18
Rural										
BMI (kg/m^2^)	−0.24	0.35	−0.15	0.15	0.08	0.15	0.08	0.12	0.08	0.09
BAZ	−0.01	0.13	−0.06	0.06	0.06	0.05	0.04	0.05	0.05	0.04
BF (%)	−0.15	0.85	−0.30	0.30	0.06	0.32	0.52	0.27	0.32	0.21
WC (cm)	−0.41	0.83	−0.40	0.40	0.24	0.36	0.25	0.30	0.25	0.22
All										
BMI (kg/m^2^)	−0.72 ***	0.21	−0.28 **	0.09	0.17	0.09	0.03	0.07	0.10	0.06
BAZ	−0.23 **	0.08	−0.08 *	0.03	0.06 *	0.03	0.01	0.03	0.03	0.02
BF (%)	−1.91 ***	0.46	−0.49 **	0.16	0.31	0.18	0.31	0.18	0.14	0.13
WC (cm)	−2.01 ***	0.57	−0.72 **	0.23	0.44 *	0.22	−0.03	0.18	0.19	0.15

BMI: Body Mass Index; BAZ: Body Mass Index-for age Z-score; BF: Body fat; WC: Waist circumference; Pedometer counts in thousand steps; Adjusted for age, sex, area of residence, energy intake, ethnicity, household income and parental education; Significant coefficient at * *p* < 0.05; ** *p* < 0.01; *** *p* < 0.001.

## 4. Discussion

This study focused on two factors, namely low level of physical activity and high sedentary behaviour, which are known risk factors for overweight and obesity in children. We found that physical activity of urban children was negatively associated with BMI, BAZ, %BF, and WC. Children with higher physical activity level were more likely to have lower BMI, BAZ, %BF as well as WC than their less physically active counterparts. This is in accordance with the study of Chaput *et al.* [27] that reported that physical activity as assessed by accelerometer had negative correlation with BMI, WC and %BF of children. Another study assessed physical activity by pedometer and found that physical activity was inversely associated with children’s BMI percentile [28]. The negative association of physical activity with %BF and WC in the present study was also consistent with the findings of Su *et al.* [29] among Malaysian adolescents aged 13 years, as well as the Duncan *et al.* [18] study of children aged 5 to 12 years in New Zealand. However, the negative association of physical activity and BMI, BAZ, %BF, and WC was not found in rural children. We hypothesise that the significant association we found in the overall models may be driven by the significant inverse relationship among urban children. Regardless of the different assessment methods used to measure physical activity in children, previous studies consistently reported that physical activity was inversely related to obesity as measured by BMI and BAZ [9,30].

The present study found that screen time was significantly associated with BAZ but not with BMI and %BF. Laurson *et al.* [28] did not find a significant correlation between screen time and BMI percentile. Our findings showed that children who had higher screen time were more likely to also have higher WC, a finding that was consistent with that reported by Kang *et al.* [31]. In contrast, we found that other non-screen sedentary activities were not related to body weight, BF and WC in children. To our knowledge there were no others studies conducted on the association between non-screen sedentary activities and childhood obesity. Sedentary activities, including both screen time and non-screen time, was not associated with BMI, WC and %BF among our population, which is consistent with findings reported by Colley *et al.* [32] and Kwon *et al.* [33]. However, a recent review paper reported that increased sedentary time is correlated to higher BMI, WC, %BF as well as higher risk of obesity [34].

The inter-relationship between physical activity, screen time and obesity is unclear. BMI of young children at 6 years of age might significantly influence their BMI later at ages 8, 10 and 14 years [35]. The adiposity of children is associated with the physical activity level of children [36]. Screen time positively predicts BMI but negatively predicts physical activity for children aged 8 and 10 years [35]. Many other studies have reported that obesity or body fatness of children were associated with physical inactivity [37,38] and high sedentary behaviour [30]. Similarly, we found that physical activity was positively associated with all measures of obesity while screen time was inversely associated with BAZ and WC.

We found that overweight and obese children had a lower physical activity levels but similar screen time as their thin and normal weight counterparts. Obese children in Malaysia spent less time on physical activities and had longer periods of sedentary activities as compared to normal weight children [39]. However, another study reported no difference in the physical activity score and time spent on sedentary activities between normal weight with overweight and obese children [40]. Chew *et al.* [41] reported that overweight and obese children from suburban areas in Malaysia had higher television viewing and electronic gadget playing time than their normal weight counterparts.

The results of the current study raise concern over the physical activity levels and screen time of Malaysian children. The majority of Malaysian children did not meet minimal physical activity recommendations (boys: 13,000 daily steps; girls: 11,000 daily steps) and exceeded the maximal screen time of 2 h/day recommendations. The average 9708 steps/day for boys and 8339 steps/day for girls found in this study suggests that Malaysian children are less active than children in Singapore [42], the Western Pacific region [43] and several European countries [43]. A higher percentage of Malaysian children (68%) exceeded the screen time recommendations compared to children in the United States (44%) [44] and Australia (59%) [45].

In agreement with the findings of previous studies [46,47], the present study reported that boys were physically more active than girls in both BMI groups. A recent systematic review concluded that physical activity has stronger association with adiposity in boys than in girls [9] especially for light and moderate-to-vigorous physical activity rather than overall physical activity [48]. Different types and intensity of daily activities between boys and girls were highly related to their physical activity level [49]. Our study also revealed that rural children had higher daily step counts than urban children. A possible explanation for this may be that rural children had higher outdoor activities than their urban counterparts as they have more opportunities available to them living in a natural environment [50]. Outdoor activities is also likely to aid in reducing children’s screen time as well as lowering the prevalence of overweight and obesity [51].

The results of present study revealed that Malaysian children spent almost half of their sedentary time on screen activities including watching television, playing video games, and using the computer. The average screen time of children in this study was 3.1 h, which is comparable to previous studies on Malaysian adolescents [52] and Mexican children and adolescents [53]. With increased accessibility and availability of electronic devices, children spend more time engaging in screen-related sedentary behaviours such as playing on their smart phones, accessing the internet, and playing video games [54]. It has been suggested that screen time contributes to the risk of obesity [35,55,56] not only by lowering total daily energy expenditure, but also through increase in energy intake [22,57].

Arundell *et al.* [58] observed that children had decreased physical activity level but increased time spent on sedentary activities over 3 and 5 years of childhood development. Interestingly, our study found that boys had higher physical activity level simultaneously with higher screen time, in comparison to girls, which is consistent with the findings of Jago *et al.* [59]. There is a good possibility that children who engaged in active sports and activities also practises sedentary behaviour at other times [59], which suggests that sedentary behaviour do not display physical activity levels of children [10]. Higher daily physical activity is a protective factor for increased body weight and abdominal adiposity in children [60,61], hence it is essential to increase physical activity level, especially among overweight and obese children.

This is the first Malaysian study that documents physical activity level, sedentary behaviours, and achievement of physical activity and screen time recommendation in a large, nationally representative sample of children aged 7 to 12 years. Another strength is that correlates of obesity, such as household income, parental education level and energy intake, were taken into account as confounders when modelling the relationship between physical activity and obesity. In addition to multiple measures of obesity, physical activity was comprehensively studied using both pedometer and questionnaires. However, we acknowledge that self-reporting of physical activity especially sedentary activities may not be sensitive or accurate enough to capture the daily sedentary behaviour of children. In addition, data on sleep h were not collected to measure comprehensively the overall sedentary behaviour of children.

## 5. Conclusions

Overall, Malaysian children from rural areas are more physically active than their urban counterparts. Boys have both higher activity level and screen time as compared to girls. The majority of the children did not meet the minimum physical activity and exceed the maximal screen time recommendation. Physical activity and screen time, but not other sedentary activities were associated with BMI, BAZ, %BF and WC in Malaysian children. Replacement of an overall less active behaviour with moderate and vigorous physical activity may provide a viable solution to improve BMI, BAZ, %BF and WC, and hence reduce childhood obesity in Malaysia, particularly among urban children. Future studies are warranted to investigate the other risk factors that may be associated with the nutritional status of rural children in Malaysia. In addition, intervention studies are needed to establish the effectiveness of reducing obesity among children through increasing physical activity in tandem with reducing sedentary behaviours, especially screen time.
